# Effects of taurine on the growth performance, diarrhea, oxidative stress and intestinal barrier function of weanling piglets

**DOI:** 10.3389/fvets.2024.1436282

**Published:** 2024-08-07

**Authors:** Miao Zhou, Zichen Wu, Donghua Deng, Bin Wang, Xiaoling Zhou, Bingyu Zhou, Chunping Wang, Yan Zeng

**Affiliations:** ^1^College of Animal Science and Technology, Hunan Agricultural University, Changsha, China; ^2^Hunan Institute of Microbiology, Changsha, China

**Keywords:** weanling piglets, taurine, oxidative stress, intestinal health, Nrf2/HO-1 signaling pathway

## Abstract

Oxidative damage resulting from weaning stress significantly impacts the growth performance and health status of piglets. Taurine, a dietary antioxidant with diverse functions, was investigated in this study for its protective role against weaning stress-induced oxidative damage and its underlying mechanism. Forty 28-day-old male castrated weaned piglets were randomly assigned to four groups. The control group received the basal diet, while the experimental groups were fed the basal diet supplemented with 0.1, 0.2%, or 0.3% taurine over a 28-day period. *In vitro*, H_2_O_2_ was utilized to induce oxidative damage to the jejunal mucosa of piglets via IPEC-J2 cells. The results demonstrated that taurine supplementation reduced the incidence of diarrhea in piglets compared to that in the control group (*p* < 0.05); the addition of 0.2 and 0.3% taurine led to increased average daily gain and improved feed conversion efficiency in weaned piglets, showing a linear dose-response correlation (*p* < 0.05). Taurine supplementation at 0.2 and 0.3% enhanced the activities of serum CAT and GSH-Px while decreasing the levels of serum NO, XOD, GSSG, and MDA (*p* < 0.05). Moreover, it significantly elevated the levels of GSS, Trx, POD, complex I, mt-nd5, and mt-nd6, enhancing superoxide anion scavenging capacity and the hydroxyl-free scavenging rate in the livers of weaned piglets while reducing NO levels in the liver (*p* < 0.05). Additionally, 0.2 and 0.3% taurine supplementation decreased serum IL-6 levels and elevated the concentrations of IgA, IgG, and IL-10 in weaned piglets (*p* < 0.05). The levels of occludin, claudin, and ZO-1 in the jejunum mucosa of weaned piglets increased with 0.2 and 0.3% taurine supplementation (*p* < 0.05). In IPEC-J2 cells, pretreatment with 25 mM taurine for 24 h enhanced the activities of SOD and CAT; reduced the MDA content; upregulated the mRNA expression of various genes, including *ZO-1*, occludin, claudin-1, *Nrf2*, and *HO-1*; and reversed the oxidative damage induced by H_2_O_2_ exposure (*p* < 0.05). Overall, the findings suggest that the inclusion of 2 and 3% taurine in the diet can enhance growth performance, reduce diarrhea rates, ameliorate oxidative stress and inflammation, and promote intestinal barrier function in weaned piglets.

## 1 Introduction

Overall, piglet breeding plays a vital role in the process of pig breeding. The proper feeding and management of piglets directly impact key metrics such as slaughter time, rate, and weight, thus influencing the production efficiency of pig breeding (1, 2). Weaning is a critical phase in the pig growth cycle and is highly susceptible to environmental stimuli, feed quality, mycotoxins, and pathogenic microorganisms. These factors discussed may result in an overproduction of intestinal reactive oxygen species (ROS) in piglets, leading to oxidative stress. This oxidative stress can subsequently trigger inflammatory damage to the intestinal mucosa and compromise barrier function. As a consequence, piglets may experience reduced growth performance, diarrhea, and potentially fatal outcomes, thereby imposing substantial economic burdens on the swine industry (3, 4).

Oxidative stress occurs when animals release oxidative free radicals that surpass the body’s antioxidant defenses, giving rise to various physiological or pathological manifestations (5, 6). Research indicates that the weaning process disrupts the redox balance in piglets, markedly reducing the activity of antioxidant enzymes while elevating free radicals and peroxide products, essentially inducing oxidative stress (7, 8). Cells respond to oxidative stress by activating various transcription factors, including nuclear factor E2-related factor 2 (Nrf2). Nrf2 plays a crucial role in regulating cellular redox balance, facilitating the expression of protective antioxidants, and initiating phase I detoxification reactions in mammals. It stands as a key target for the body in the management of oxidative stress (9). Dietary antioxidants such as anthocyanins and Vitamin E are potent natural antioxidants capable of efficiently scavenging free radicals, thereby safeguarding cells against oxidative damage and immune stress (10, 11). Consequently, enhancing the redox status of piglets is a crucial strategy for fostering healthy development.

Supplementing piglet diets with functional components has emerged as a feasible and effective approach for mitigating oxidative stress postweaning (7, 12). Taurine, a sulfur-containing nonprotein amino acid, plays pivotal roles in cellular processes such as volume regulation, osmotic pressure adjustment, protein phosphorylation, membrane stability, bile acid metabolism, neural modulation, calcium homeostasis, and detoxification and exhibits antioxidant and anti-inflammatory properties that are crucial for maintaining animal homeostasis (13, 14). Recent research underscores the ability of taurine to alleviate various oxidative damage-related ailments and its involvement in immune system modulation, offering significant benefits to animal production (15–17). Therefore, this study aimed to assess how taurine impacts growth performance, oxidative stress levels, immune function, and intestinal barrier integrity in weaned piglets.

## 2 Materials and methods

This animal study was reviewed and approved by the Hunan Agricultural University Institutional Animal Care and Use Committee (202105). Written informed consent was obtained from the owners for the participation of their animals in this study.

### 2.1 Materials

Taurine (purity ≥99%) and hydrogen peroxide (purity 30%) were purchased from Shanghai Aladdin Biochemical Technology Co., Ltd.

### 2.2 Experimental design and diets

Forty 28-day-old healthy castrated Duroc × Landrace × Large weaned piglets were randomly divided into 4 groups with 10 replicates in each group and 1 pig in each replicate. The plants were fed in a single pen and fed for 3 days. The control group was fed a basal diet, and the experimental group was fed a diet supplemented with 0.1, 0.2% or 0.3% taurine. The test period was 28 days. The basic diet was prepared according to the NRC 2012, and the feed composition and nutritional level are shown in Table 1. The piglet house was cleaned and disinfected before the experiment. During the experiment, the animals were fed twice a day, provided food freely and allowed to drink freely. The piglets were subjected to routine immunization procedures, and their health status was carefully observed and recorded every day.

**Table 1:** Diet composition and nutrient levels (as-fed basis).

Ingredients	Content	Nutrient levels^b^	Content
Corn, %	61.72	Digestible energy, MJ/kg	14.44
Soybean meal, %	11.88	Crude protein, %	18.38
Fermented soybean meal, %	6.00	Calcium, %	0.80
Fish meal, %	5.00	Total phosphorus, %	0.68
Whey powder, %	10.00	Available phosphorus, %	0.40
Fatty powder, %	1.59	Lysine, %	1.40
CaHPO_4_, %	0.94	Methionine, %	0.50
Limestone, %	0.63	Methionine + cystine, %	0.76
NaCl, %	0.30	Threonine, %	0.90
L-Lysine HCl, %	0.55	Tryptophan, %	0.21
DL-Methionine, %	0.20		
L-Threonine, %	0.20		
Premix^a^, %	1.00		
Total, %	100.00		

a The premix provided the following per kg of diet: V_A_ 10500 IU, V_D3_ 3,000 IU, V_E_ 22.5 IU, V_K3_ 3.0 mg, pantothenic 15 mg, riboflavin 7.5 mg, folic acid 1.5 mg, niacinamide 30.0 mg, thiamine 3.0 mg, VB_6_ 4.5 mg, biotin 0.12 mg, V_B12_ 0.03 mg, Zn 100 mg, Fe 104 mg, Mn 4.0 mg, Cu 6.0 mg, I 0.3 mg, and Se 0.3 mg.

b The digestible energy and amino acids were calculated according to the NRC (2012), and the analysis of crude protein, calcium, total phosphorus, and available phosphorus was conducted using the reference method provided by the AOAC (2006).

### 2.3 Sample collection

After 12 h of fasting, the anterior vena cava of the piglets was collected on the morning of the 29th day of the experimental period. The whole blood was placed in an ordinary vacuum blood collection vessel. After standing at room temperature for 30 min, the mixture was centrifuged at 845 g and 4°C for 10 min, after which the supernatant was separated in a 1.5 mL EP tube. After quick freezing with liquid nitrogen, the samples were stored at −80°C. The patients were randomly anesthetized with pentobarbital sodium (40 mg/kg) and killed by bloodletting. The thoracic cavity and abdominal cavity were opened, and the liver and jejunum were separated. An appropriate amount of liver was removed from the frozen tube at the fixed position and stored at −80°C after quick freezing with liquid nitrogen. Approximately 20 cm of middle jejunum tissue was removed, and the tissue was rinsed with normal saline. The mucosal layer was scraped with a slide, collected in a cryopreservation tube for quick freezing with liquid nitrogen, and then stored at −80°C.

### 2.4 Growth performance

Piglets were weighed on an empty stomach on the mornings of day 1 and day 29 of the experiment. During the experiment, daily feed intake and residual feed weight were recorded to calculate the average daily gain (ADG), average daily feed intake (ADFI) and feed-to-gain ratio (F/G). The diarrhea of the piglets was observed and recorded at 5 PM every day, and the diarrhea rate (%) = number of piglets with diarrhea × 100/28.

### 2.5 Serum parameters

Serum catalase (CAT), glutathione peroxidase (GSH-Px), superoxide dismutase (SOD), total antioxidant capacity (T-AOC), superoxide anion scavenging capacity, hydroxyl radical scavenging rate, nitric oxide (NO), xanthine oxidase activity (XOD), glutathione (GSH), oxidized glutathione (GSSG), malondialdehyde (MDA) and peroxidase (POD) contents were detected by biochemical kits produced by Nanjing Jianchen Bioengineering Institute, China. Serum immunoglobulin A (IgA), immunoglobulin G (IgG), immunoglobulin M (IgM), interleukin-4 (IL-4), interleukin-10 (IL-10), interleukin-1β (IL-1β), interleukin-6 (IL-6), and tumor necrosis factor-α (TNF-α) were detected by ELISA kits, which were purchased from Jiangsu Enzyme Immunoassay Industry Co., Ltd., China. All procedures were performed in strict accordance with the kit instructions.

### 2.6 Analysis of hepatic oxidative status

The levels of glutathione synthase (GSS), thiredoxin (Trx), NO, superoxide anion scavenging capacity, hydroxyl radical scavenging rate, POD, complex I, mitochondrially encoded NADH dehydrogenase 5 (mt-ND5) and mitochondrially encoded NADH dehydrogenase 6 (mt-ND6) were measured. All operations were carried out in strict accordance with the instructions of the kit produced by Nanjing Jianchen Biotechnology Institute, China.

### 2.7 Analysis of jejunal mucosal barrier proteins

The levels of claudin-1, tight junction protein 1 (ZO-1) and Occludin in the jejunal mucosa were detected by ELISA kits purchased from Jiangsu Enzyme Immunoassay Industry Co., Ltd., China. All operations were carried out strictly according to the instructions of the kit.

### 2.8 Cell culture

Porcine jejunal epithelial cells (IPEC-J2) were cultured in complete DMEM/F12 supplemented with 10% FBS (OPCEL, Inner Mongolia Opcel Biotechnology Co., Ltd., BS1101) and 1% penicillin–streptomycin (Gibco, 15140-122) in a 5% CO_2_ cell incubator at 37°C. When the cells reached 80% confluence, they were washed twice with sterile PBS and then subcultured or treated with drugs. The cell seeding ratios for culture vessels of various sizes were as follows: 5 × 10^6^ cells/cm^2^ for 100 mm cell culture dishes; 9 × 10^5^ cells/cm^2^ for 6-well cell culture plates; and 3 × 10^4^ cells/cm^2^ for 96-well cell culture plates.

### 2.9 Selection of the daidzein concentration

To determine the optimal taurine concentration, IPEC-J2 cells were seeded in 96-well cell culture plates with six replicates per treatment. After reaching approximately 80% confluency, the cells were subjected to two washes with sterile PBS. Subsequently, 100 μL of DMEM/F12 medium with various concentrations of taurosulfonate (0, 10, 25, 50, and 100 mM, supplemented with 100 μg/mL streptomycin and 100 U/mL penicillin) was added to each well for a 24 h incubation period. After another round of PBS washes, the model group received 100 μL of the treatment medium containing 0.6 mM H_2_O_2_ according to the methods of Li et al. (18), while the blank group was treated with the same volume of the treatment medium without H_2_O_2_. Cell viability assessment was conducted using the CCK8 assay after 1 h of incubation.

### 2.10 Measurements of T-AOC, SOD, and CAT activity and MDA content

The cells were cultured and treated in 6-well plates. Cells cultured to 80% confluence were preincubated with the treatment medium, either with or without 25 mM taurine, for 24 h. Subsequently, the cells were exposed to 0.6 mM H_2_O_2_ for 1 h, harvested and analyzed following the operational guidelines of the biochemical kits for T-AOC (G0115W), SOD (G0101W), CAT (G0105W), and MDA (G0109W) provided by Suzhou Grandis Biotechnology Co., Ltd., China.

### 2.11 RT-qPCR

Cells were categorized into four groups based on the presence or absence of 25 mM taurine and 0.6 mM H_2_O_2_ as per the culture method outlined in *section 2.10*. Upon completion of cell culture, the cells were harvested using a cell scraper, followed by total RNA extraction using the TRIzol method. Subsequently, the mRNA expression levels of the *Nrf2*, *HO-1*, *CAT*, *SOD*, *ZO-1*, occludin, and claudin-1 genes were analyzed through quantitative real-time PCR. The porcine-specific primers utilized in this study were custom-designed (Table 2). The PCR cycles and relative expression assays adhered to the methodology outlined in our prior research study conducted by Yin et al. (19).

**Table 2:** Primers used for gene expression analysis by real-time PCR.

Gene	Primer sequence (5′–3′)	Product length, bp
*Nrf2*	F: GACCTTGGAGTAAGTCGAGA	103
	R: GGAGTTGTTCTTGTCTTTCC	
*HO-1*	F: GAGAAGGCTTTAAGCTGGTG	74
	R: GTTGTGCTCAATCTCCTCCT	
*SOD1*	F: GAAGACAGTGTTAGTAACGG	93
	R: CAGCCTTGTGTATTATCTCC	
*CAT*	F: CCTGCAACGTTCTGTAAGGC	72
	R: GCTTCATCTGGTCACTGGCT	
*ZO-1*	F: TTGATAGTGGCGTTGACA	126
	R: CCTCATCTTCATCATCTTCTAC	
*Claudin-1*	F: GCATCATTTCCTCCCTGTT	97
	R: TCTTGGCTTTGGGTGGTT	
*Occludin*	F: CAGTGGTAACTTGGAGGCGTCTTC	103
	R: CGTCGTGTAGTCTGTCTCGTAATGG	
*β-actin*	F: CTGCGGCATCCACGAAACT	147
	R: AGGGCCGTGATCTCCTTCTG	

*Nrf2*, nuclear factor-erythroid2-related factor 2; *HO-1*, heme oxygenase-1; *SOD1*, superoxide dismutase 1; *CAT*, catalase; *ZO-1*, zonula occludens 1.

### 2.12 Statistical analysis

In animal experiments, a single-factor random design is utilized, with each pig serving as a designated statistical unit. Normally distributed data were subjected to single-factor ANOVA, along with pairwise group comparisons via the Duncan method. Polynomial contrast analysis is implemented to evaluate the linear and quadratic impacts of the additives. A nonparametric Kruskal–Wallis test was performed if the data deviated from a normal distribution, followed by a *post hoc* Dunn test for intergroup comparisons. The graphs were generated using GraphPad Prism 9.0.0 software. Statistical significance was determined at the *p* < 0.05 level. The results are presented as the means and standard errors of the means (SEMs).

## 3 Results

### 3.1 Growth performance

The impact of taurine on the growth performance of weanling piglets is detailed in Table 3. Compared with those in the control group, the inclusion of 0.2 and 0.3% taurine in the diet significantly enhanced the average final weight and ADG of the piglets while markedly reducing the F/G. The final weight and ADG of the piglets increased proportionally to the taurine concentration, while the F/G ratio decreased linearly. Moreover, supplementing the diets of weaned piglets with 0.1, 0.2%, or 0.3% taurine led to a significant reduction in diarrhea incidence, with both linear and quadratic effects.

**Table 3:** Effect of taurine on the growth performance of weanling piglets.

Item	Taurine inclusion level, %				SEM	*p*-value		
	0	0.1	0.2	0.3		Treatment	Linear	Quadratic
Initial body weight, kg	6.79	6.71	6.73	6.73	0.07	0.981	0.829	0.776
Final body weight, kg	12.65^b^	13.00^b^	14.02^a^	14.48^a^	0.21	0.002	0.000	0.871
ADG, kg/day	167.52^b^	179.84^b^	208.29^a^	221.22^a^	6.13	0.003	0.000	0.974
ADFI, kg/day	432.19	439.44	453.44	481.44	9.09	0.267	0.061	0.566
F/G	2.58^a^	2.45^a^	2.19^b^	2.19^b^	0.04	0.000	0.000	0.324
Diarrhea rate, %	11.43^a^	1.34^b^	0.00^b^	3.93^b^	1.07	0.000	0.001	0.000

ADG, average daily gain; ADFI, average daily feed intake; F/G, the ratio of feed intake to gain. The mean values of the same row of data without the same superscript letter were significantly different at the *p*-value (*n* = 10).

### 3.2 Serum antioxidant parameters

The analysis presented in Table 4 demonstrates that supplementing the diets of weaned piglets with 0.1, 0.2, and 0.3% taurine resulted in an increase in the serum GSH levels in comparison to those in the control group. The inclusion of 0.2 and 0.3% taurine led to elevated serum CAT activity and reduced levels of serum NO, XOD, GSSG, and MDA in the weaned piglets. Moreover, the introduction of 0.3% taurine enhanced the activities of GSH-Px and SOD in piglet serum and increased the T-AOC and POD, superoxide anion scavenging capacity, and hydroxyl radical scavenging rate. The study also revealed a linear increase in CAT, GSH-Px, SOD, T-AOC, superoxide anion scavenging capacity, the hydroxyl radical scavenging rate, GSH, and POD with increasing taurine concentration. Conversely, there were linear decreases in the levels of NO, XOD, GSSG, and MDA.

**Table 4:** Effect of taurine on serum antioxidant indices in weanling piglets.

Item	Taurine inclusion level, %				SEM	*p*-value		
	0	0.1	0.2	0.3		Treatment	Linear	Quadratic
CAT, nmol/min/mL	15.72^b^	15.95^b^	24.16^a^	24.14^a^	1.25	0.001	0.000	0.934
GSH-Px, nmol/min/mL	700.93^b^	703.66^b^	841.29^a^	870.08^a^	25.77	0.010	0.002	0.727
SOD, U/mL	197.46^b^	209.04^b^	248.75^b^	388.57^a^	22.23	0.000	0.000	0.017
T-AOC, μmol Trolox/mL	0.12^b^	0.12^b^	0.14^b^	0.19^a^	0.01	0.000	0.000	0.017
Superoxide anion scavenging capacity, %	16.81^b^	16.96^b^	17.35^b^	18.64^a^	0.24	0.010	0.002	0.120
Hydroxyl radical scavenging rate, %	3.46^b^	3.55^b^	3.78^ab^	3.97^a^	0.07	0.022	0.003	0.619
NO, μmoL/mL	0.36^a^	0.33^a^	0.25^b^	0.24^b^	0.02	0.006	0.001	0.670
XOD, U/mL	5.79^a^	5.67^a^	3.23^b^	2.22^b^	0.45	0.000	0.000	0.251
GSH, U/mL	0.08^c^	0.14^b^	0.17^b^	0.24^a^	0.02	0.000	0.000	0.604
GSSG, nmol/mL	3.61^a^	3.18^b^	3.14^b^	2.71^c^	0.11	0.001	0.000	0.955
MDA, nmol/mL	13.00^a^	11.26^a^	8.41^b^	6.97^b^	0.69	0.000	0.000	0.800
POD, U/mL	7.25^b^	8.52^b^	8.71^b^	12.99^a^	0.60	0.000	0.000	0.010

CAT, catalase; GSH-Px, glutathione peroxidase; SOD, superoxide dismutase; T-AOC, total antioxidant capacity; NO, nitric oxide; XOD, xanthine oxidase activity; GSH, glutathione; GSSG, oxidized glutathione; MDA, malondialdehyde; POD, peroxidase. The mean values of the same row of data without the same superscript letter were significantly different at the *p*-value (*n* = 10).

### 3.3 Liver antioxidant parameters

The findings presented in Table 5 illustrate the impact of taurine on the antioxidant capacity of the liver of weanling piglets. Compared with those in the control group, dietary supplementation with 0.1, 0.2, and 0.3% taurine resulted in increased levels of liver GSS, Trx, POD, and mt-ND5; enhanced superoxide anion clearance capacity; and an elevated hydroxyl-free clearance rate while reducing the liver NO levels in the weaned piglets. Moreover, administering 0.2 and 0.3% taurine led to increased levels of liver complex I and mt-ND6 in weanling piglets. The present study also revealed a linear increase in GSS, Trx, superoxide anion scavenging capacity, the hydroxyl radical scavenging rate, POD, complex I, mt-ND5, and mt-ND6 with increasing taurine concentrations and a linear decrease in NO levels.

**Table 5:** Effect of taurine on antioxidant indices in the liver of weanling piglets.

Item	Taurine inclusion level, %				SEM	*p*-value		
	0	0.1	0.2	0.3		Treatment	Linear	Quadratic
GSS, U/g	0.39^d^	0.43^c^	0.52^b^	0.58^a^	0.02	0.000	0.000	0.297
Trx, ng/g	745.17^d^	935.07^c^	1052.05^b^	1226.66^a^	46.05	0.000	0.000	0.697
NO, μmoL/mgProt	0.19^a^	0.18^b^	0.16^c^	0.10^d^	0.01	0.000	0.000	0.000
Superoxide anion scavenging capacity, %	16.22^d^	17.37^c^	18.47^b^	19.02^a^	0.29	0.000	0.000	0.080
Hydroxyl radical scavenging rate, %	3.41^c^	3.70^b^	3.80^b^	4.22^a^	0.08	0.000	0.000	0.384
POD, U/mgProt	3.37^d^	4.95^c^	6.07^b^	7.67^a^	0.42	0.000	0.000	0.962
Complex I, pg/g	7.89^c^	8.31^c^	9.00^b^	9.62^a^	0.19	0.000	0.000	0.590
mt-ND5, pg/g	4.86^d^	6.34^c^	7.24^b^	8.72^a^	0.37	0.000	0.000	0.987
mt-ND6, pg/g	3.64^c^	3.85^c^	5.34^b^	6.22^a^	0.29	0.000	0.000	0.115

CAT, catalase; GSH-Px, glutathione peroxidase; SOD, superoxide dismutase; T-AOC, total antioxidant capacity; NO, nitric oxide; XOD, xanthine oxidase activity; GSH, glutathione; GSSG, oxidized glutathione; MDA, malondialdehyde; POD, peroxidase. GSS, glutathione synthase; Trx, thiredoxin; NO, nitric oxide; POD, peroxidase; Complex I, mt-ND5, mitochondrially encoded NADH dehydrogenase 5; mt-ND6, mitochondrially encoded NADH dehydrogenase 6. The mean values of the same row of data without the same superscript letter were significantly different at the *p*-value (*n* = 10).

### 3.4 Serum immune parameters

Table 6 shows that the supplementation of 0.1, 0.2, and 0.3% taurine in the diet of weaned piglets led to a significant reduction in the serum IL-6 levels and a notable increase in the IgA and IgG levels compared to those in the control group. Moreover, the addition of 0.2 and 0.3% taurine significantly elevated the serum IL-10 level, whereas the inclusion of 0.3% taurine notably increased the serum IL-4 and IgM levels. Notably, the levels of IL-4, IL-10, IgA, IgG, and IgM exhibited both linear and quadratic growth patterns in correlation with increases in the taurine concentration, while the level of IL-6 decreased linearly.

**Table 6:** Effect of taurine on serum immune indices in weanling piglets.

Item	Taurine inclusion level, %				SEM	*p*-value		
	0	0.1	0.2	0.3		Treatment	Linear	Quadratic
IL-4, pg/mL	22.35^b^	27.16^b^	30.73^b^	51.54^a^	3.17	0.000	0.000	0.020
IL-10, pg/mL	35.86^c^	49.92^bc^	70.73^b^	118.83^a^	8.74	0.000	0.000	0.038
IL-1β, pg/mL	232.92	256.12	314.57	190.78	22.22	0.287	0.730	0.116
IL-6, pg/mL	346.53^a^	284.70^b^	202.42^c^	92.72^d^	25.57	0.000	0.000	0.160
TNF-α, pg/mL	80.40	71.28	78.28	80.97	4.16	0.864	0.833	0.525
IgA, μg/mL	263.28^c^	345.53^b^	293.92^bc^	476.46^a^	25.80	0.000	0.000	0.015
IgG, μg/mL	3.67^c^	6.26^b^	6.03^bc^	13.55^a^	1.08	0.000	0.000	0.010
IgM, mg/mL	7.27^b^	8.10^b^	8.04^b^	15.44^a^	0.97	0.000	0.000	0.000

IgA, immunoglobulin A; IgG, immunoglobulin G; IgM, immunoglobulin M; IL-4, interleukin-4; IL-10, interleukin-10; IL-1β, interleukin-1β; IL-6, interleukin-6; TNF-α, tumor necrosis factor-α. The mean values of the same row of data without the same superscript letter were significantly different at the *p*-value (*n* = 10).

### 3.5 Jejunal mucosal barrier proteins

Table 7 shows that, in comparison to those in the control group, supplementation of the diets of weaned piglets with 0.1, 0.2, and 0.3% taurine led to a notable increase in occludin levels in the jejunal mucosa. Moreover, the inclusion of 0.2 and 0.3% taurine markedly elevated the levels of claudin and ZO-1. Concurrently, occludin, claudin, and ZO-1 levels exhibited both linear and quadratic increases proportional to the increase in taurine concentration.

**Table 7:** Effects of taurine on jejunal mucosal immune indices in weanling piglets.

Item	Taurine inclusion level, %				SEM	*p*-value		
	0	1	2	3		Treatment	Linear	Quadratic
Claudin-1, ng/g	636.88^c^	682.24^c^	1038.14^b^	1469.29^a^	88.18	0.000	0.000	0.000
ZO-1, ng/g	714.90^c^	756.72^c^	879.10^b^	1258.34^a^	55.84	0.000	0.000	0.000
Occludin, ng/g	33.49^d^	47.28^c^	66.02^b^	133.75^a^	10.01	0.000	0.000	0.000

ZO-1, zonula occludens 1. The mean values of the same row of data without the same superscript letter were significantly different at the *p*-value (*n* = 10).

### 3.6 The concentration of taurine in IPEC-J2 cells

**Figure 1:**
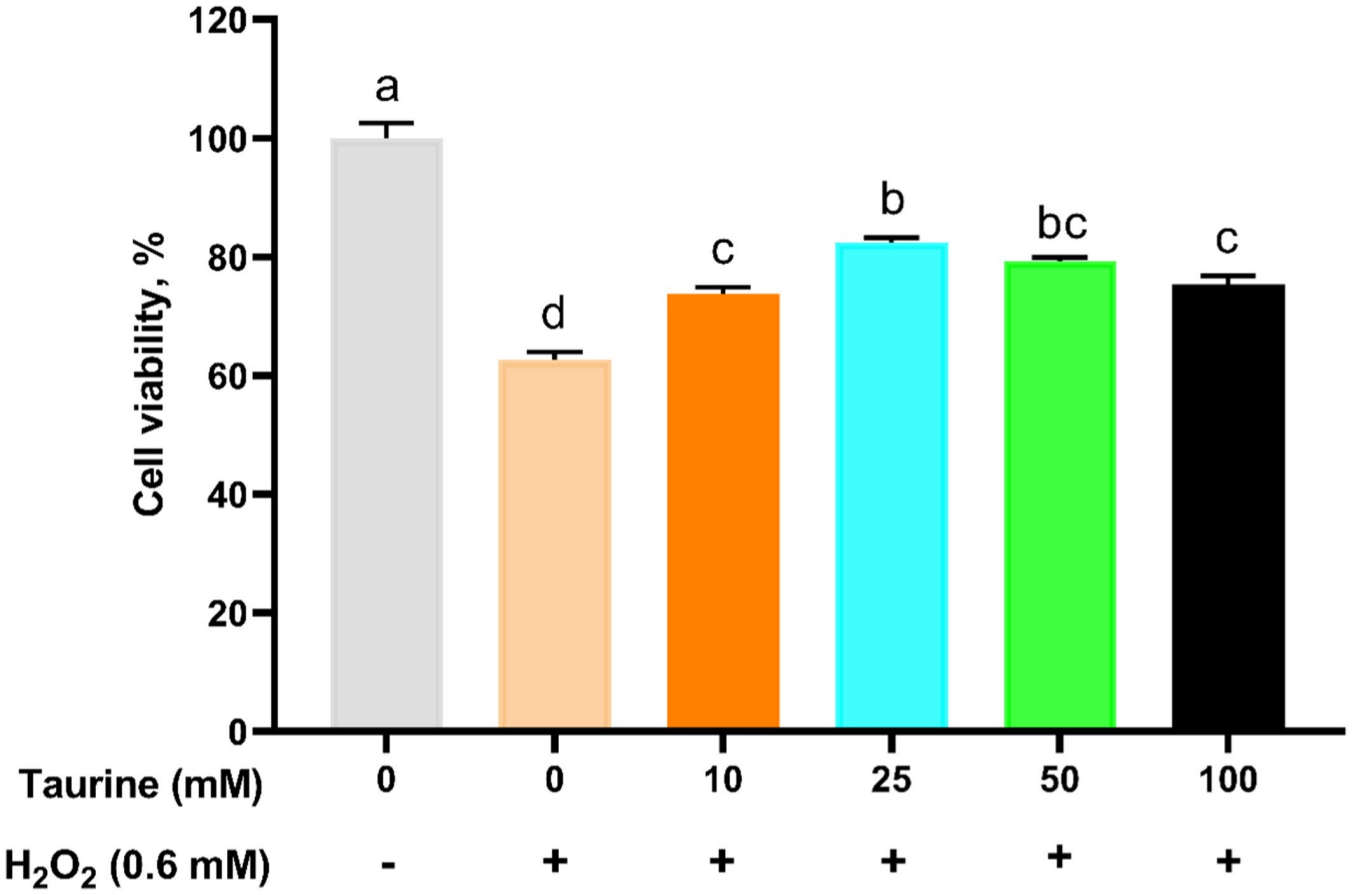
Effect of taurine on IPEC-J2 cell viability. IPEC-J2 cells were cultured in 96-well plates and preincubated with various concentrations of taurine (ranging from 0 to 100 mM) for 24 h, followed by exposure to 0.6 mM H_2_O_2_ for 1 h. The results, presented as the mean ± standard error of the mean (SEM), *n* = 6, indicate the values as a percentage relative to the untreated group with neither H_2_O_2_ nor taurine. Significant differences (*p* < 0.05) are denoted by the distinct letters a, b, c, and d. The following abbreviations were used: IPEC-J2, porcine intestinal epithelial cells; H_2_O_2_, hydrogen peroxide.

To determine the optimal treatment parameters for taurine. The cells were pretreated with various taurine concentrations for 24 h and subsequently either exposed or not exposed to 0.6 mM H_2_O_2_ for 1 h. Cell viability assessments revealed that the highest viability rate occurred in the 25 mM taurine treatment group (Figure 1). Consequently, we selected 25 mM as the optimal concentration for all subsequent experiments.

### 3.7 SOD activity, CAT activity, T-AOC and MDA content in H_2_O_2_-treated IPEC-J2 cells

**Figure 2:**
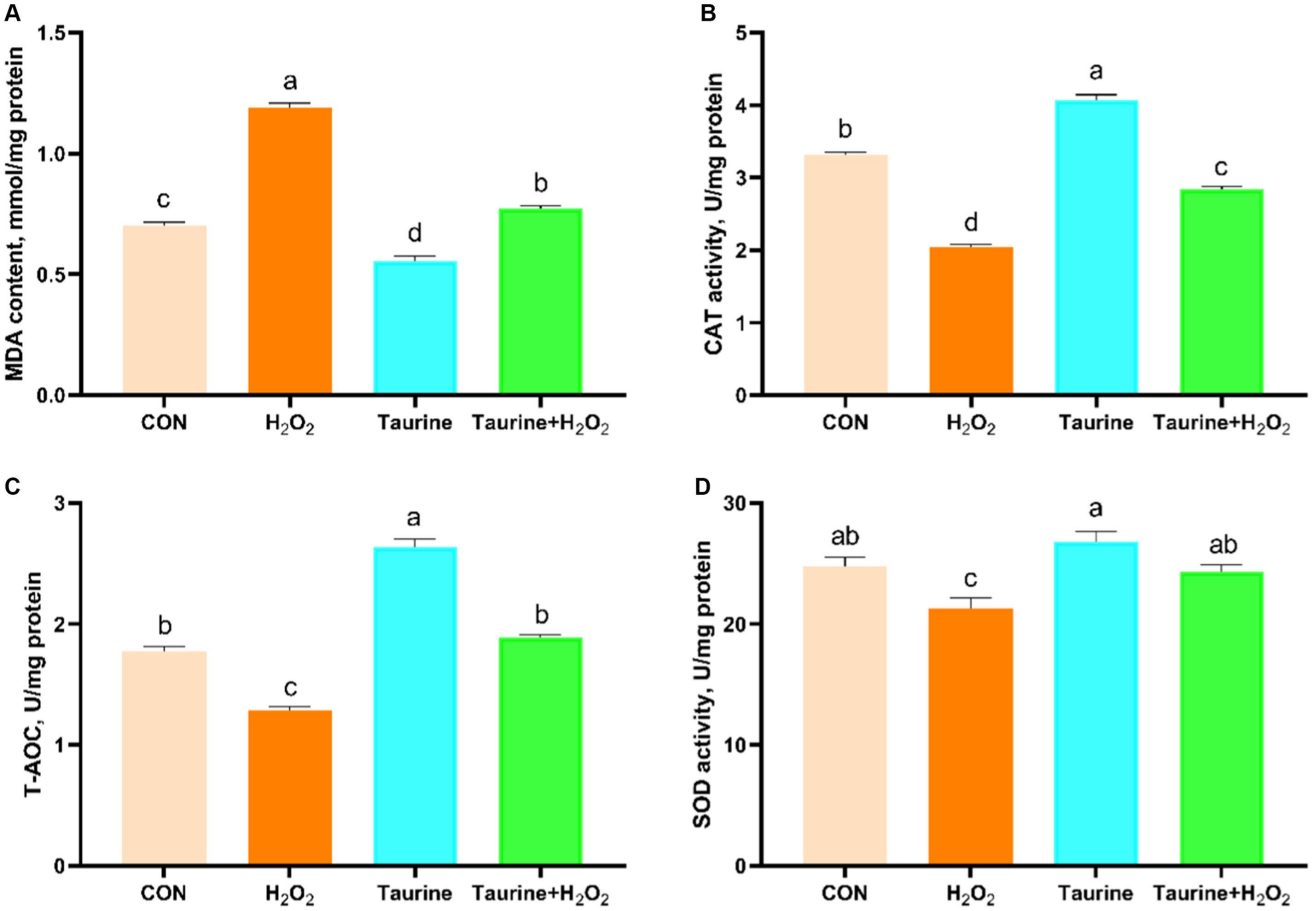
Effect of taurine on antioxidant enzymes and MDA in IPEC-J2 cells. **(A)** MDA content. **(B)** CAT activity. **(C)** Total antioxidant capacity. **(D)** SOD activity. IPEC-J2 cells were cultured in 6-well plates and pretreated with either 25 mM taurine or no taurine for 24 h, followed by exposure to 0.6 mM H_2_O_2_ or no H_2_O_2_ for 1 h. The results are presented as the mean ± standard error of the mean (SEM), *n* = 6. Statistical significance, denoted by *p* < 0.05, is indicated by values assigned unique alphabetical labels (a, b, c). IPEC-J2, porcine intestinal epithelial cells; H_2_O_2_, hydrogen peroxide; MDA, malondialdehyde; CAT, catalase; T-AOC, total antioxidant capacity; SOD, superoxide dismutase.

This study focused on investigating the impact of taurine on antioxidant enzyme activity and MDA content in IPEC-J2 cells exposed to catalase. Biochemical kits (Figure 2) were utilized for analysis. Exposure to H_2_O_2_ significantly decreased the activities of CAT and SOD, as well as the T-AOC, while notably increasing the MDA content in the cells compared to those in the control group. As expected, 25 mM pretreatment for 24 h not only significantly boosted the antioxidant capacity of the cells but also ameliorated the oxidative stress injury caused by H_2_O_2_ exposure. This pretreatment led to a considerable increase in CAT and SOD activities and T-AOC, coupled with a reduction in MDA content.

### 3.8 Effect of taurine on the expression of genes involved in the Nrf2 signaling pathway in IPEC-J2 cells

**Figure 3:**
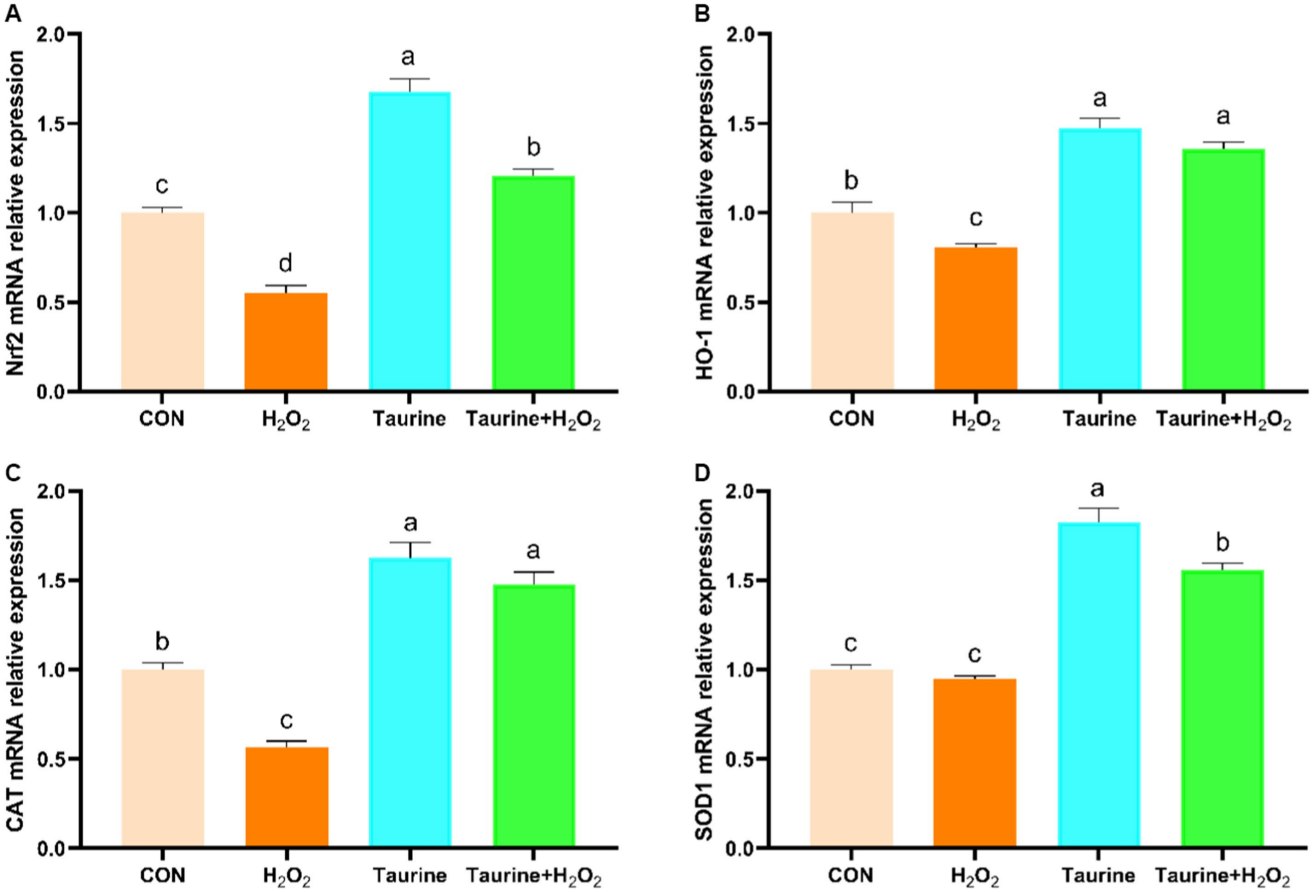
Effect of taurine on the expression of genes involved in the Nrf2 signaling pathway in IPEC-J2 cells. **(A)** *Nrf2* mRNA expression relative to that of β-actin. **(B)** *HO-1* mRNA expression relative to that of β-actin. **(C)** *CAT* mRNA expression relative to that of β-actin. **(D)** *SOD1* mRNA expression relative to that of β-actin. IPEC-J2 cells were cultured in 6-well plates and pretreated with either 25 mM taurine or no taurine for 24 h, followed by exposure to 0.6 mM H_2_O_2_ or no H_2_O_2_ for 1 h. The results are presented as the mean ± standard error of the mean (SEM), *n* = 6. Statistical significance, denoted by *p* < 0.05, is indicated by values assigned unique alphabetical labels (a, b, c). IPEC-J2, porcine intestinal epithelial cells; H_2_O_2_, hydrogen peroxide; MDA, malondialdehyde; CAT, catalase; T-AOC, total antioxidant capacity; SOD, superoxide dismutase.

The Nrf2 signaling pathway serves as a crucial regulatory mechanism in combating oxidative stress. Our study explored the impact of pretreatment with taurine on the expression levels of Nrf2 pathway genes in H_2_O_2_-exposed IPEC-J2 cells (Figure 3). Compared to those in the control group, the expression of the *Nrf2*, *HO-1* and *CAT* genes in the H_2_O_2_-treated group was notably lower. Conversely, pretreatment with 25 mM taurine for 24 h substantially upregulated the expression of *the Nrf2*, *HO-1* and *CAT* genes, leading to a significant amelioration of oxidative damage caused by H_2_O_2_ exposure.

### 3.9 Effect of taurine on barrier function-related gene expression in IPEC-J2 cells exposed to H_2_O_2_

**Figure 4:**
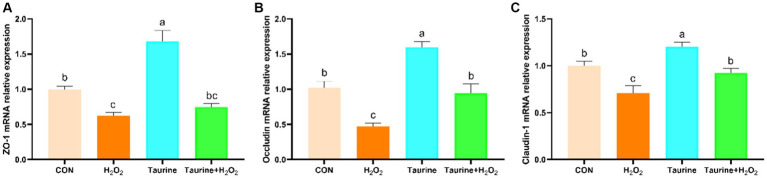
Effect of taurine on barrier function-related gene expression in IPEC-J2 cells. **(A)** *ZO-1* mRNA expression relative to that of β-actin. **(B)** Occludin mRNA expression relative to that of β-actin. **(C)** Claudin-1 mRNA expression relative to that of β-actin. IPEC-J2 cells were cultured in 6-well plates and pretreated with either 25 mM taurine or no taurine for 24 h, followed by exposure to 0.6 mM H_2_O_2_ or no H_2_O_2_ for 1 h. The results are presented as the mean ± standard error of the mean (SEM), *n* = 6. Statistical significance, denoted by *p* < 0.05, is indicated by values assigned unique alphabetical labels (a, b, c). IPEC-J2, porcine intestinal epithelial cells; H_2_O_2_, hydrogen peroxide; ZO-1, zonula occludens 1.

The impact of taurine pretreatment on the expression levels of tight junction-related genes in IPEC-J2 cells exposed to H_2_O_2_ is illustrated in Figure 4. Compared to those in the control group, H_2_O_2_ exposure markedly decreased the expression of the *ZO-1*, occludin, and claudin-1 genes. Conversely, pretreatment with 25 mM taurine for 24 h significantly upregulated the expression of the *ZO-1*, occludin, and claudin-1 genes, effectively reversing the downregulation induced by H_2_O_2_.

## 4 Discussion

Weanling stress leads to diarrhea, reduced growth performance, compromised intestinal function, and other harmful effects, resulting in notable economic losses for the pig breeding industry (20, 21). This study aimed to investigate the potential benefits of taurine on the growth performance, oxidative stress, and intestinal barrier of weaned piglets. Currently, supplementing diets with antioxidants is an effective strategy for mitigating oxidative stress and enhancing the growth performance and health status of weaned piglets (7). Furthermore, past research has indicated that antioxidants can serve as alternatives to antibiotics in facilitating smooth weaning for piglets (22). Our findings revealed that the addition of taurine to the diets of weaned piglets, particularly at the 0.2 and 0.3% levels, significantly increased both the average final weight and daily gain of the piglets while reducing the feed-to-meat ratio. Consistent with the findings of a recent study by Wang et al. (23), taurine supplementation at 0.2 and 0.4% notably enhanced the growth performance of weaned piglets. Moreover, previous studies have suggested that taurine could mitigate the adverse impacts of LPS on the growth performance of piglets and broilers (16, 24). During the weaning process, piglets often experience diarrhea due to diverse external stressors and incomplete development. Each year, a considerable number of weaned piglets succumb to or are culled because of weaning stress-related diarrhea, resulting in significant losses in the breeding sector (3, 25). Our research showed that taurine effectively decreased the rate of diarrhea in weaned piglets compared to that in the control group. Subsequent studies revealed that taurine improved the oxidative stress levels and intestinal barrier function of piglets, further corroborating the potential of taurine as an antioxidant substitute for feed antibiotics to ameliorate the growth performance and diarrhea of weaned piglets.

Weanling stress is not only closely related to potential changes in the immune system and intestinal barrier function but also results in severe oxidative stress (26). The body’s redox reactions underlie various biochemical pathways and cellular functions, primarily reliant on the delicate equilibrium between the oxidative and antioxidant systems. An imbalance due to excessive ROS production or inadequate ROS scavenging by antioxidants induces oxidative stress, leading to cell apoptosis, tissue damage, metabolic disorders, inflammation, diarrhea, and reduced production performance in piglets (27, 28). Studies by Wen et al. (29) highlighted a notable increase in malondialdehyde (MDA) levels in the blood of weaned piglets, along with decreased SOD and GSH-Px activities. Taurine can scavenge reactive oxygen species and mitigate lipid peroxidation. The levels of SOD, GSH-Px, and MDA in blood and tissues are common biomarkers of oxidative stress. MDA is a metabolite of autolipid peroxidation, and an increase in GSH and SOD levels reflects an increase in host antioxidant capacity. Our research demonstrated that taurine supplementation enhances serum GSH-Px activity and GSH levels and improves superoxide anion and hydroxyl radical scavenging capacities while reducing NO, XOD, and GSSG serum levels. Moreover, oxidative stress is intricately linked to ferroptosis, where both GSH-Px and GSH play crucial roles as regulators by facilitating the reduction of ROS (30, 31). In conclusion, our findings align with previous research suggesting that taurine enhances the serum antioxidant capacity of weaned piglets.

The liver, an essential organ, performs various vital functions, including detoxification, metabolism, bile secretion, and immune defense (32). It exhibits high sensitivity to exogenous substances, rendering it susceptible to oxidative stress induced by drugs, viruses, or toxins. Weanling stress can trigger oxidative stress in the liver of piglets (33, 34). Previous research has demonstrated that weaning decreases the activities of SOD, CAT, and GSH-PX in the liver of piglets (35). Numerous studies support the protective role of taurine in liver injury. In our study, taurine increased the levels of GSS, Trx, POD, complex I, mt-nd5, and mt-nd6 in the livers of weaned piglets. It enhanced superoxide anion scavenging capacity and the hydroxyl free radical scavenging rate and notably reduced NO levels in the liver. Wu et al. (36) reported that taurine inhibits the increase in MDA and decrease in antioxidant enzyme activity in rat livers induced by aflatoxin B1. Shi et al. (37) reported the efficacy of taurine in alleviating liver oxidative damage from oxidized fish oil in young catfish. Our findings align with these studies, suggesting the potential of taurine to enhance the antioxidant capacity of the livers of weaned piglets.

Weanling stress commonly results in compromised immune function and an inflammatory response (38). During weaning, piglets are exposed to bacteria, toxins, and antigens from the intestinal cavity, which can infiltrate tissues, organs, and bloodstream through the submucosa, leading to disruptions in the intestinal immune system and triggering inflammation (39, 40). Excessive proinflammatory cytokines can exacerbate damage to intestinal integrity and epithelial function. Researchers studied the gene expression of inflammatory cytokines in weanling piglets and noted a significant increase in IL-6 levels in the intestinal tract, as did the levels of IL-1β and TNF-α (41, 42). Taurine exhibits anti-inflammatory properties and contributes significantly to managing cardiovascular diseases and metabolic inflammatory conditions such as diabetes mellitus and nonalcoholic fatty liver disease (15, 43, 44). Neutrophils are the primary contributors to tissue damage in mastitis. Studies have shown that taurine can mitigate neutrophil aggregation, hinder cxcl2 expression, and alleviate mastitis caused by *Streptococcus uberis* in dairy cows (45). Additionally, research has demonstrated that taurine supplementation can ameliorate liver cell swelling and inflammatory infiltration in piglets afflicted with mycotoxins, reducing the serum levels of the proinflammatory factors IL-1β, IL-6, IL-8, and TNF-α (46). Our study corroborates these findings, showing that incorporating taurine into the diets of weaned piglets increases the serum levels of IgA, IgG, IgM, IL-4, and IL-10 while reducing IL-6 levels. This finding suggested that taurine plays a crucial role in immune regulation, counteracting immune function impairment in weaned piglets due to weanling stress.

The jejunum serves as a vital organ for nutrient absorption from the external environment and plays a crucial role in communication with both the internal and external environments (47, 48). Due to its sensitivity to internal and external factors, the jejunum can experience oxidative stress, leading to abnormalities in its morphology and structure, damage to barrier functions, and compromised digestion and absorption capabilities (49). Consequently, oxidative damage to the jejunal mucosa is closely linked to the development of conditions such as growth retardation and diarrhea in livestock and poultry (12, 50). In this study, the supplementation of weanling piglets with taurine significantly elevated the levels of claudin, ZO-1, and occludin in the jejunal mucosa. Previous research by Shi et al. (37) demonstrated that incorporating taurine into a diet containing oxidized fish oil markedly upregulated intestinal *ZO-1* and *ZO-2* mRNA expression in catfish seedlings. Additionally, Zhao et al. (24) reported that taurine could increase the protein expression of ZO-1, occludin, and claudin-1 in the colons of piglets challenged with LPS. These findings align with those of the present study, reinforcing the notion that taurine has the potential to alleviate Weanling stress-induced impairment of intestinal barrier function.

The intestinal structure is primarily composed of villi and crypts, which are enveloped by a single layer of columnar epithelial cells (51, 52). These absorptive cells, constituting 90 to 95% of epithelial cells, feature apical microvilli and house various enzymes responsible for the breakdown and absorption of sugars and proteins (53–55). Furthermore, intestinal epithelial cells act as a crucial interface between immune cells and environmental agents, assisting in the detection and response to food, symbiotic bacteria, and pathogens to protect against pathogen invasion (56, 57). Thus, intestinal epithelial cells play a pivotal role in intestinal function. Oxidative stress has the potential to harm the intestinal mucosa. In this investigation, IPEC-J2 were utilized to replicate the oxidative damage observed in the intestinal mucosa due to weanling stress in piglets through exposure to H_2_O_2_. The presence of H_2_O_2_ triggers the generation of ROS and results in lipid damage, leading to the production of lipid degradation biomarkers (MDA). The present study revealed that pretreatment of IPEC-J2 cells with 25 mM taurine for 24 h significantly enhanced the activities of antioxidant enzymes and reduced the MDA content. Additionally, taurine pretreatment for 24 h substantially mitigated the oxidative damage induced by H_2_O_2_ exposure in IPEC-J2 cells, reversing the decreases in SOD and CAT activities and the increase in MDA content caused by H_2_O_2_ exposure. SOD is widely distributed in the mitochondrial matrix of animal cells and plays a crucial role in scavenging oxygen free radicals to protect intestinal mucosal cells from oxidative damage, making it a vital antioxidant in the body (58). Furthermore, taurine pretreatment for 24 h reversed the downregulation of genes encoding barrier proteins induced by H_2_O_2_, underscoring the role of taurine in ameliorating H_2_O_2_-induced mucosal injury.

The Nrf2 signaling pathway, which is crucial for the oxidative stress response, plays a vital role in maintaining the body’s redox homeostasis. Excessive ROS production can impede Nrf2 expression, leading to a redox imbalance (59, 60). Our study investigated the expression of Nrf2 pathway-related genes in IPEC-J2 cells using qPCR. We observed that taurine significantly enhanced *Nrf2* and *HO-1* gene expression, reversing the downregulation induced by H_2_O_2_ exposure. HO-1, a target of Nrf2, directly regulates HO-1 promoter activity through Nrf2, exhibiting potent antioxidant effects by scavenging ROS and defending against harmful substances such as peroxides and free radicals (61, 62). Taurine pretreatment also upregulated *SOD* and *CAT* gene expression in IPEC-J2 cells. Consistent with previous research, taurine shows promise for alleviating oxidative stress. Researchers have demonstrated that taurine, through *Nrf2* activation, protects pig mammary gland epithelial cells from oxidative stress induced by H_2_O_2_ (63). Furthermore, studies by Wang et al. (64) revealed that taurine could mitigate the downregulation of *Nrf2* and *HO-1* induced by 5-fluorouracil, ameliorating mucosal inflammation in mouse jejunum and colon tissues. Our findings support the potential of taurine as a dietary supplement for combating oxidative stress.

## 5 Conclusion

This study demonstrated that taurine enhances the growth performance of weaned piglets, reduces the incidence of diarrhea, alleviates oxidative stress, and mitigates inflammatory responses associated with weaning. Taurine supplementation can upregulate the expression of genes and proteins related to tight junctions in the jejunal intestinal epithelium. Additionally, taurine has the potential to ameliorate oxidative damage in intestinal epithelial cells through the Nrf2/HO-1 pathway. In conclusion, taurine has emerged as a promising dietary supplement for mitigating oxidative damage in weanling piglets.

## Data availability statement

The raw data supporting the conclusions of this article will be made available by the authors, without undue reservation.

## Ethics statement

The animal studies were approved by Hunan Agricultural University Institutional Animal Care and Use Committee (202105). The studies were conducted in accordance with the local legislation and institutional requirements. Written informed consent was obtained from the owners for the participation of their animals in this study.

## Author contributions

MZ: Data curation, Formal analysis, Investigation, Methodology, Software, Writing – original draft, Writing – review & editing. ZW: Formal analysis, Investigation, Methodology, Software, Writing – original draft, Writing – review & editing. DD: Formal analysis, Investigation, Software, Writing – original draft, Writing – review & editing. BW: Investigation, Software, Writing – original draft, Writing – review & editing. XZ: Investigation, Software, Writing – original draft, Writing – review & editing. BZ: Investigation, Software, Writing – original draft, Writing – review & editing. CW: Investigation, Software, Writing – original draft, Writing – review & editing. YZ: Conceptualization, Investigation, Project administration, Resources, Writing – original draft, Writing – review & editing.
